# Delineating the heterogeneity of embryo preimplantation development using automated and accurate morphokinetic annotation

**DOI:** 10.1007/s10815-023-02806-y

**Published:** 2023-06-10

**Authors:** Nir Zabari, Yoav Kan-Tor, Yuval Or, Zeev Shoham, Yoel Shufaro, Dganit Richter, Iris Har-Vardi, Assaf Ben-Meir, Naama Srebnik, Amnon Buxboim

**Affiliations:** 1 School of Computer Science and Engineering, The Hebrew University of Jerusalem, The Edmond J. Safra Campus, 9190416 Jerusalem, Israel; 2 The Center for Interdisciplinary Data Science Research, The Hebrew University of Jerusalem, The Edmond J. Safra Campus, Givat Ram, 9190401 Jerusalem, Israel; 3 Department of Obstetrics and Gynecology, Division of Reproductive Endocrinology and Infertility, Kaplan Hospital, Rehovot, Israel; 4 Infertility and IVF Unit, Helen Schneider Hospital for Women, Rabin Medical Center, Beilinson Hospital, Petach Tikva, Israel; 5 The IVF Unit Gyn/Obs, Soroka University Medical Center, Beer-Sheva, Israel; 6 Faculty of Health Sciences, Ben-Gurion University of the Negev, Beer-Sheva, Israel; 7 Department of Obstetrics and Gynecology, Hadassah Medical Center - Hebrew University of Jerusalem, Jerusalem, Israel; 8 Infertility and IVF Unit, Hadassah Hebrew University Hospital, Jerusalem, Israel; 9 The Alexander Silberman Institute of Life Sciences, The Hebrew University of Jerusalem, The Edmond J. Safra Campus - Givat Ram, 9190401 Jerusalem, Israel; 10 In Vitro Fertilization Unit, Department of Obstetrics and Gynecology, Shaare Zedek Medical Center, 9103102 Jerusalem, Israel; 11 The Alexender Grass Center for Bioengineering, The Hebrew University of Jerusalem, The Edmond J. Safra Campus, Givat Ram, 9190401 Jerusalem, Israel

**Keywords:** Machine learning, Assisted reproductive technologies, IVF, Embryo morphokinetic classification, Automated morphokinetic annotation

## Abstract

**Purpose:**

Our objective was to design an automated deep learning model that extracts the morphokinetic events of embryos that were recorded by time-lapse incubators. Using automated annotation, we set out to characterize the temporal heterogeneity of preimplantation development across a large number of embryos.

**Methods:**

To perform a retrospective study, we used a dataset of video files of 67,707 embryos from four IVF clinics. A convolutional neural network (CNN) model was trained to assess the developmental states that appear in single frames from 20,253 manually-annotated embryos. Probability-weighted superposition of multiple predicted states was permitted, thus accounting for visual uncertainties. Superimposed embryo states were collapsed onto discrete series of morphokinetic events via monotonic regression of whole-embryo profiles. Unsupervised K-means clustering was applied to define subpopulations of embryos of distinctive morphokinetic profiles.

**Results:**

We perform automated assessment of single-frame embryo states with 97% accuracy and demonstrate whole-embryo morphokinetic annotation with R-square 0.994. High quality embryos that had been valid candidates for transfer were clustered into nine subpopulations, as characterized by distinctive developmental dynamics. Retrospective comparative analysis of transfer versus implantation rates reveals differences between embryo clusters as marked by poor synchronization of the third mitotic cell-cleavage cycle.

**Conclusions:**

By demonstrating fully automated, accurate, and standardized morphokinetic annotation of time-lapse embryo recordings from IVF clinics, we provide practical means to overcome current limitations that hinder the implementation of morphokinetic decision-support tools within clinical IVF settings due to inter-observer and intra-observer manual annotation variations and workload constrains. Furthermore, our work provides a platform to address embryo heterogeneity using dimensionality-reduced morphokinetic descriptions of preimplantation development.

**Supplementary Information:**

The online version contains supplementary material available at 10.1007/s10815-023-02806-y.

## Introduction

Owing to the inherent biological heterogeneity in the developmental potential of embryos, decreasing the risks that are associated with multiple pregnancy while shortening time to pregnancy relies on transferring the embryo(s) of the highest developmental quality. To address this important need, various assisted reproductive technologies (ARTs) have been developed [1]. Embryos that are generated via in vitro fertilization (IVF) and harbor chromosomal abnormalities that are associated with a decreased potential to implant and generate a live birth can be screened via preimplantation genetic testing for aneuploidy (PGT-A) and for structural chromosomal rearrangements (PGT-SR) [2]. However, PGT is invasive and requires obtaining cellular biopsies from the embryos. In addition, false negative assessments may arise due to insufficient genetic amplification and chromosomal mosaicism [3, 4].

**Fig. 1:**
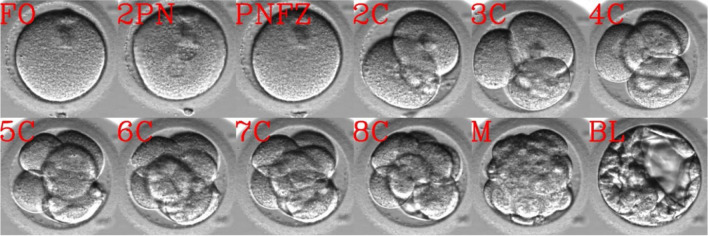
Preimplantation embryo development. Time lapse visualization of the developmental states of an embryo are demonstrated during preimplantation development. Abbreviations. FO, fertilized oocyte; 2PN, two pronuclei; PNFZ, pronuclei faded zygote; 2C – to – 8C, two – to – eight cells; M, morula; BL, blastocyst

Complementary to PGT, various non-invasive technologies have been developed during the past decade for assessing embryo quality and developmental potential in real time [5]. Such approaches are based on the assumptions that embryo quality can be linked with certain metabolic markers, which can be assessed by probing molecular signatures in the spent culture medium [6–9]. Other methodologies relay on the mechanical changes in the viscoelastic properties of preimplantation embryos that are associated with high developmental quality and can be defined by measuring the stress–strain relationships under applied load [10]. However, predicting embryo quality based on its visualization made the highest impact in the clinic. The first protocols scored embryo potential based on certain morphological grading criteria of specific developmental stages [11–14]. The utilization of time-lapse incubation systems in IVF clinics provided dynamic and continuous visualization of the embryos as they monotonically advance between the states of preimplantation developmental while maintaining the embryos under optimal culture conditions (Fig. 1). Using these video recordings, the embryos can be characterized by the specific time points from fertilization at which discrete developmental events occur [15]. These so-called morphokinetic events are defined by the transition between embryo states and include times of pronuclei appearance (tPNa) and fading (tPNf), the cleavage of two-to-eight blastomeres (tN, *N* = 1 to 8), the compaction of the morula (tM), and start of blastulation (tSB) [14, 16]. The generation of large datasets of morphokinetically annotated and clinically labeled embryos facilitated the development of classification algorithms that predict the potential for embryo implantation [17, 18] and live birth [19–21]. Parallel efforts took advantage of the size of the available time-lapse datasets to train convolutional neural network (CNN) based classifiers that assess embryo potential using the raw video files in an unbiased annotation-independent manner [22, 23]. However, training such deep learning models is challenging due to the size of the video files (~ 100’s Mb), which would require a sufficiently large sample number [24, 25].

Morphokinetic evaluation of the developmental potential proved highly efficient in de-selecting for transfer poor quality embryos. However, annotation by trained embryologists of all the embryos from each oocyte collection cycle is time-consuming. Moreover, manual morphokinetic annotation can introduce inter-observer and intra-observer variations. Multiple retrospective studies report “…generally good, although not optimal…” inter-observer and intra-observer agreement between embryologists [26–29]. These limitations stimulated the development of automated classifiers that extract the developmental stages of the embryos and the time of morphokinetic events [30–34]. To support the automated and standardized morphokinetic assessment of embryo developmental potential with high accuracy that will allow clinical implementation, we used a large dataset of manually-annotated and clinically-labeled embryo video files, and trained a CNN classifier to infer the developmental states that are recorded in each frame. For each individual frame, we allowed the classification of multiple developmental states in a manner that the confidence in the prediction of these states is weighted (the sum of all probabilities is one). By allowing this superposition of multiple predicted states at different probabilities, we included in the model the morphokinetic potential uncertainties which may improve accuracy. We then constrained chronological development as appears by the time-lapse frame-wise series of developmental states to obtain the morphokinetic profiles of the embryos. Indeed, we predict the developmental states of the single frames with 97% accuracy and the series of morphokinetic profiles of the embryos from time of pronuclei appearance to start of blastulation with R-squared coefficient of determination 0.994 as validated across 1918 test set embryos. Using our automated classifier, we provide unparalleled temporal statistics of preimplantation development of 67,707 embryos. Focusing on 14,159 high-quality embryos that would correspond to valid candidates for transfer, we apply unsupervised clustering into nine distinctive cohorts. We define distinctive patterns of early and late morphokinetics and reveal cluster-specific correlations with the rate of embryo transfer and the rate of embryo implantation. Our work thus provides a standardized platform for assessing the developmental potential of pre-implantation embryos. By supporting single embryo transfer policies, our work is expected to decrease the medical risks that are associated with multiple pregnancies and shorten time to pregnancy.

## Methods

### Dataset

Here we used a previously assembled database [23]. In short, we assembled a large dataset of 67,707 video files of preimplantation embryo development that were recorded on eleven time-lapse incubation systems (EmbryoScope Time-Lapse System, Vitrolife) located in four medical centers. The dataset includes 20,253 embryos that were morphokinetically manually-annotated by trained embryologists adhering to established protocols as we reported previously [23]. Time-lapse images were recorded with an average 18 min time interval for 3-to-6 days. At each time point, seven Z-stack frames were recorded; however, only the central focal plane was used here. The embryos in the dataset were randomly distributed such that ~ 20% of the frames were dedicated to serve as an uncontaminated test set (Table 1). The remaining frames were divided between a train set (~ 85%) and a test set (~ 15%). Frames belonging to individual embryos were not shared between the test set and the train/validation set.

**Table 1:** Number of train, validation, and test set frames across embryo states

Embryo state	1C	PN	2C	3C	4C	5C	6C	7C	≥ 8C	M	≥ SB
Train set frames	545,995	220,455	310,304	59,280	268,836	72,835	70,580	69,282	370,065	132,471	303,459
Validation set frames	95,742	39,350	54,895	10,320	47,439	12,887	12,473	12,168	65,227	23,456	53,860
Test set frames	148,436	59,904	83,867	16,400	74,324	19,520	19,351	19,384	124,027	48,847	104,758

The number of embryos that were transferred to the uterus 3 days (Day-3), 4 days (Day-4), and 5 days (Day-5) from the time of fertilization is provided per medical center in Table 2. We specify the number of embryos with positive and negative known implantation data (KID) as well as unknown KID embryos. The latter corresponds to multiple transfers in which the identity of the implanted embryos is not known.

**Table 2:** Number of Day-3, Day-4, and Day-5 transferred embryos afros medical centers H1 to H4. KID-P, known implantation data positive; KID-N, KID-negative; KID-UN, KID-unknown; Day-3, 66–74 h from fertilization; Day-4, 75–110 h from fertilization; Day-5, 111–125 h from fertilization

	Day-3 transfers			Day-4 transfers			Day-4 transfers		
KID	KID-P	KID-UK	KID-N	KID-P	KID-UK	KID-N	KID-P	KID-UK	KID-N
H1	61	319	477	6	324	26	15	104	47
H2	92	275	838	23	256	157	24	124	169
H3	132	200	468	26	38	71	166	187	308
H4	196	198	122	34	29	2	114	73	48

### Embryo-state frame labeling

The morphokinetic events characterize the preimplantation dynamics of the embryos and are not a property of the individual time-lapse frames. Hence, we first converted the manually-annotated morphokinetic profiles of the embryos into the so-called developmental embryo state labels of each individual frame. Given the monotonic nature of preimplantation development, the conversion of the manually annotated morphokinetic profiles into embryo state labels was performed in straightforward manner as specified in Table 3. Frames that overlap the manually annotated morphokinetic events and the frames that were recorded just after were excluded from the train set. Notably, here, we discriminate between $FO$ and $PNFZ$ embryo states despite being morphologically-identical. Hence, $FO$ and $PNFZ$ frames were re-labeled one cell ($1C$) for the purpose of network training.

**Table 3:** Conversion rules for setting the embryo states based on the morphokinetic events. FO, fertilized oocyte; $tPNa$ and $tPNf$, time of PN appearance and fading; ${t}_{N}$, time of N blastomeres cleavage event; ${t}_{M}$, time of Morula compaction; ${t}_{SB}$, time of start-of-blastulation; PN, pronuclei; PNFZ, PN-fading zygote

Morphokinetic event	Embryo state
$t<tPNa$	$FO$
$tPNa\le t<tPNf$	$PN$
$tPNf\le t<{t}_{2}$	$PNFZ$
${t}_{N}\le t<{t}_{N+1}$	$NC, 2\le N\le 7$
${t}_{8}\le t<{t}_{M}$	$8{C}^{+}$
${t}_{M}\le t<{t}_{SB}$	$M$
${t}_{SB}\le t$	$S{B}^{+}$
$t={t}_{n} or {t}_{n+1}$	Frames discarded from train-set

### Frame preprocessing

The Embryoscope time-lapse incubator (Vitrolife) records 8-bit grayscale images that are composed of 500 × 500 grayscale pixels. To decrease dimensionality, a 256 × 256 pixels region of interest (ROI) of the embryos in each frame was cropped using a U-net segmentation network as we reported previously [23]. In addition, all train-set frames were further augmented by applying [$90^\circ , 180^\circ , 270^\circ ]$ rigid rotations, horizontal flipping, and vertical flipping.

### Inference of the frame-wise embryo-state probability vector and the embryo probability matrix

To infer the developmental states of the embryo as visualized in each individual frame, we trained a ResNet18 CNN [35], using the train, validation, and test sets of the time-lapse frames labeled by the embryo developmental states as described above. Since these are grayscale image files, we modified the first convolutional layer to input a single channel instead of three. The CNN model was implemented using TorchVision in PyTorch with a categorical cross-entropy loss function, and optimized using Rectified Adam (RAdam). A 0.0005 learning rate was set, reaching convergence within ten epochs.

**Fig. 2:**
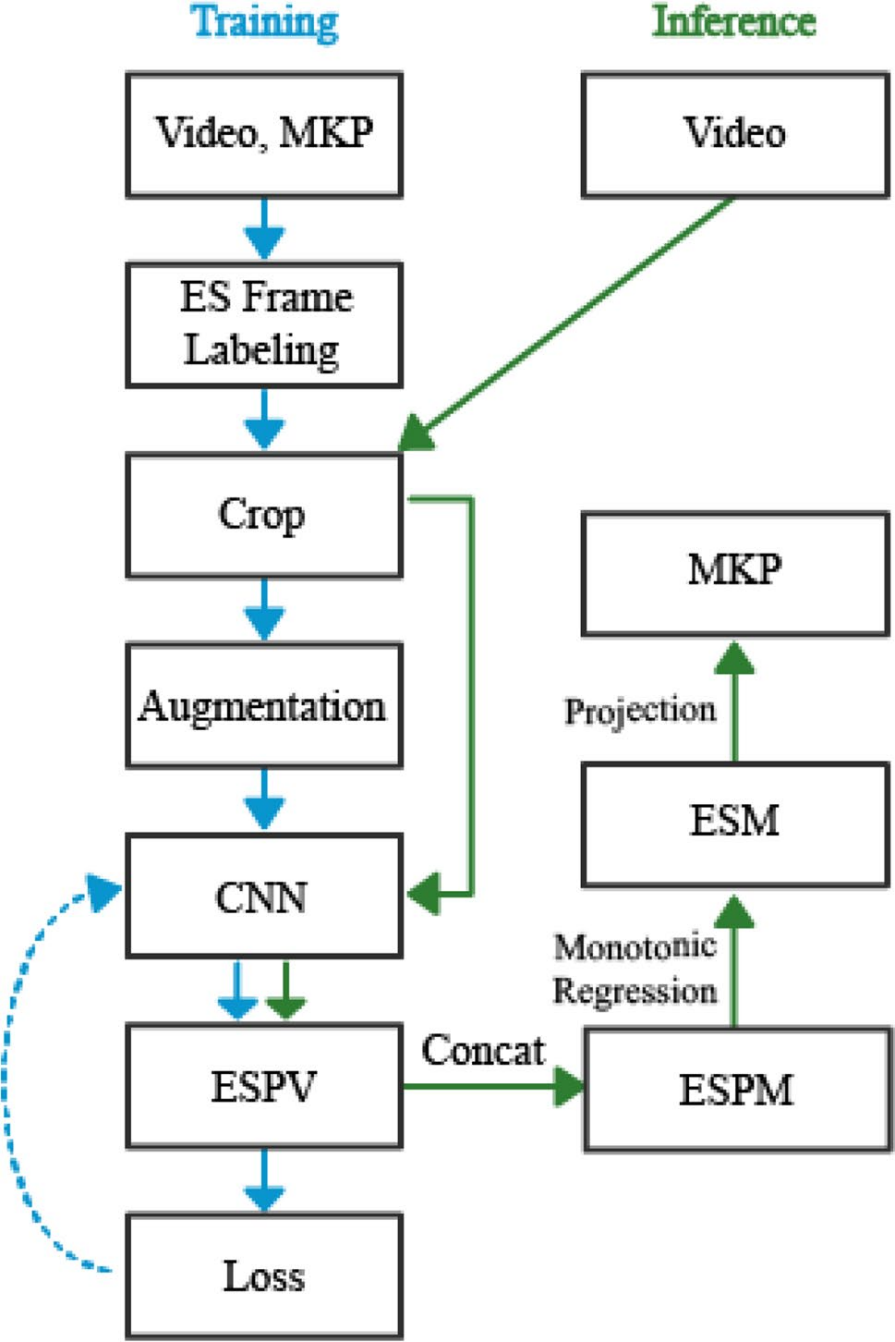
Training a CNN model for performing automated morphokinetic annotation of embryo video files. Training (blue): Morphokinetically manually-annotated input frames are labeled by the recorded developmental states and used for training of a CNN classifier of the ESPV as illustrated. Inference (green): For each embryo, the corresponding ESPVs are generated, concatenated (ESPM), and projected via monotonic regression (ESM) to extract the MKP. CNN, convolutional neural network; ESPV, embryo state probability vector; ESPM, embryo state probability matrix; ESM, embryo state matrix; MKP, morphokinetic profile

Let us consider the time-lapse sequence of size $n$ of a given embryo, where ${t}_{i}$ is the time from fertilization of frame $i=1,\dots ,n$. The classifier infers the probability to find the embryo at any of the developmental states, from 1C to BL, as obtained by the eleven output neurons (Fig. 2). In this manner, the weighted and superimposed developmental state of frame $i$ is defined by the embryo state probability vector (*ESPV*) of the output neuron coordinates:

$${ESPV}_{i}\in {\left[\mathrm{0,1}\right]}^{11}$$

**Fig. 3:**
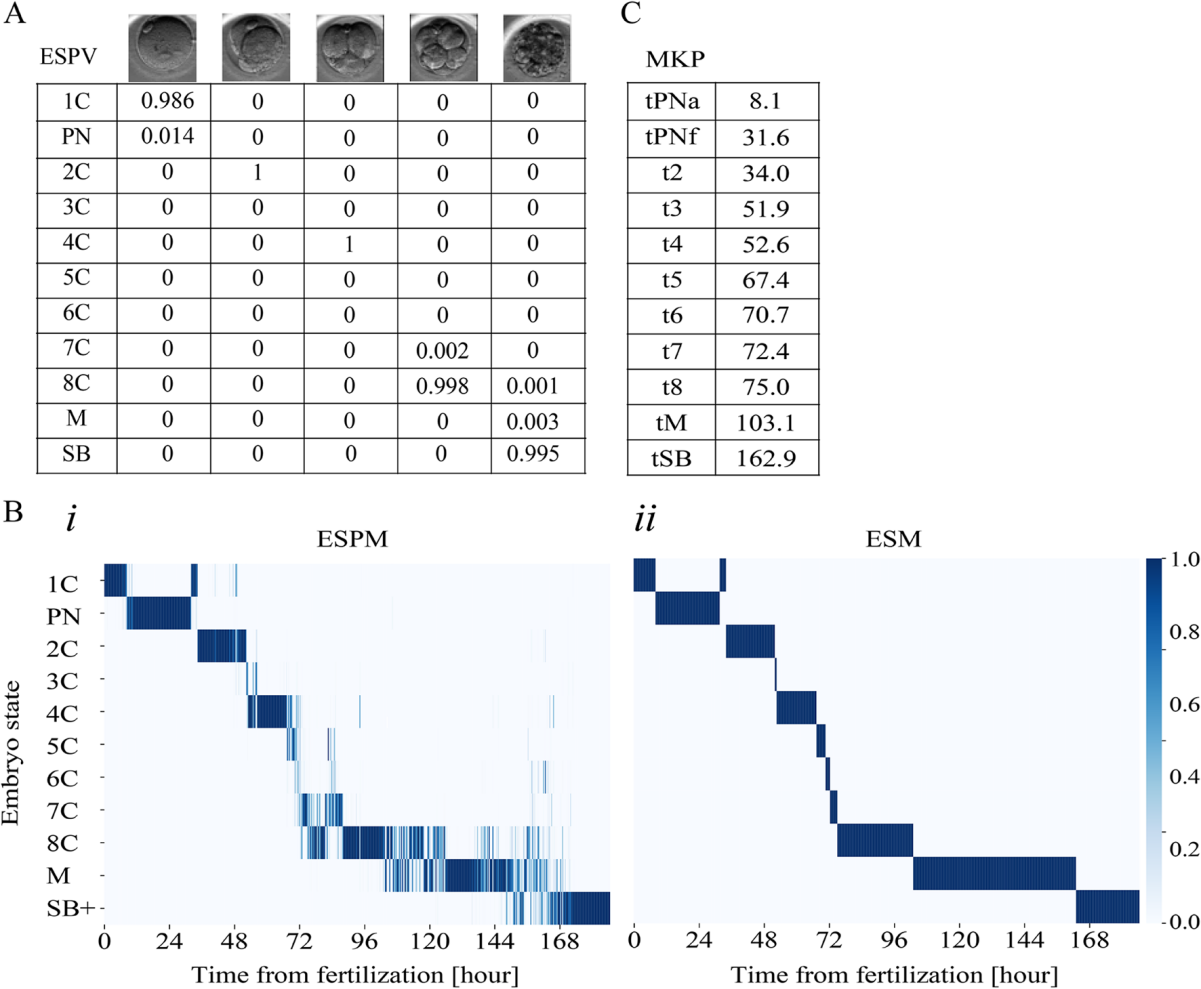
Automated morphokinetic annotation: ESPV to MKP. Automated morphokinetic annotation is demonstrated for a representative embryo. **A** The ESPM is generated via chronological concatenation (left to right) of the ESPV columns. **B** (i) A heatmap presentation of the ESPM superimposes multi-state distributions of the embryo at each time point (columns). (ii) The superimposed states are projected onto a single state at each time point via monotonic regression as appear by the ESM. The FO and the PNFZ share a one-cell (1C) label owing to their indistinguishable morphology. **C** The MKP’s are set by discrete events of the temporal transitions between embryo states in the ESM. MKP, morphokinetic profiles; ESPV, embryo state probability vector; ESPM, embryo state probability matrix; ESM, embryo state matrix

The *ESPVs* of five representative snapshots at ascending order are shown in Fig. 3A. To obtain a probability-weighted whole-embryo dynamic representation of preimplantation development, we define the embryo-state probability matrix (*ESPM*) by concatenating all the *ESPVs* of the embryo in a chronological order (Fig. 3B(i)).

$${ESPM}_{i,j}\in {\left[\mathrm{0,1}\right]}^{11\times n}$$

### Automated annotation of the morphokinetic events

To extract the discrete time points of the morphokinetic events, the uncertainty in the developmental states of the embryo, which are formulated by the ESPM, should first be projected onto discrete temporal states. Hence, we project ESPM onto $\widehat{ESM}\in {\left\{\mathrm{0,1}\right\}}^{11\times n}$ as follows:

$${\widehat{ESM}}_{i,j}=\left\{\begin{array}{l}0,\;\; \underset{{j}^{^{\prime}}}{\mathrm{arg\;max}}\left({ESPM}_{i,{j}^{^{\prime}}}\right)\ne j\\ 1,\;\; \underset{{j}^{^{\prime}}}{\mathrm{arg\;max}}\left({ESPM}_{i,{j}^{^{\prime}}}\right)=j\end{array}\right.$$

Hence, $\widehat{ESM}$ provides a discrete description of the temporal states of the embryo. However, it does not satisfy the monotonicity of preimplantation development. To this end, we apply a weightless isotonic regression of $\widehat{ESM}$:

$$\underset{{y}_{i,j}\in {\left[\mathrm{0,1}\right]}^{11\times n}}{\mathrm{min}}\sum_{j=1}^{11}{\left({y}_{i,j}-{\widehat{ESM}}_{i,j}\right)}^{2} s.t. \underset{j}{\mathrm{arg max}}\left({y}_{i,j}\right)\le \underset{j}{\mathrm{arg max}}\left({y}_{{i}^{^{\prime}},j}\right)\leftrightarrow {t}_{i}\le {t}_{{i}^{^{\prime}}}$$

The binary and discrete matrix $\widehat{y}\in {\left[\mathrm{0,1}\right]}^{11\times n}$ is defined by:

$${\widehat{y}}_{i,j}=round\left({y}_{i,j}\right)$$

We recall that FO and PNFZ are two embryo states that share the 1C morphological label which is used here. Hence, we expect to obtain at least two temporally-separated regions in the ESPM of value 1C (see Fig. 3B(i)) that will be propagated to $\widehat{ESM}$ but converged in $\widehat{y}$. Based on trial-and-error, we find that FO and PNFZ are best captured by the earliest and the latest 1C regions in $\widehat{y}$, respectively. Hence, we replace the FO and PNFZ time regions (first row) in $\widehat{y}$ and obtain the binary and developmentally-monotonic embryo state matrix $ESM\in {\left\{\mathrm{0,1}\right\}}^{11\times n}$ (Fig. 3B(ii)).

With increasing $i$ ($ESM$ columns), there are up to eleven transitions between the embryo states that correspond to the morphokinetic events of that embryo, which we extract in a straightforward manner. The time of the morphokinetic event $i$ is thus defined by the transition from embryo state $j$ to the consecutive one (most frequently $j+1$). In the case of direct equal cleavage from $m$ cells, $m>1$, to $\left(m+2\right)$ cells, the morphokinetic events $\left(m+1\right)C$ and $\left(m+2\right)C$ will converge. The vector of time points of the morphokinetic events is the automatically annotated morphokinetic profile of that embryo (Fig. 3C).

### Unsupervised clustering

Unsupervised clustering of the embryos’ morphokinetic profiles was performed via *K*-means with Euclidian metric using the scikit-learn Python package. The number of clusters was determined to optimize the interplay between minimizing cluster number and maximizing variance as presented by the elbow plot (Fig. 8A).

## Results

### Inference uncertainty of the frame-wise embryo states

**Fig. 4:**
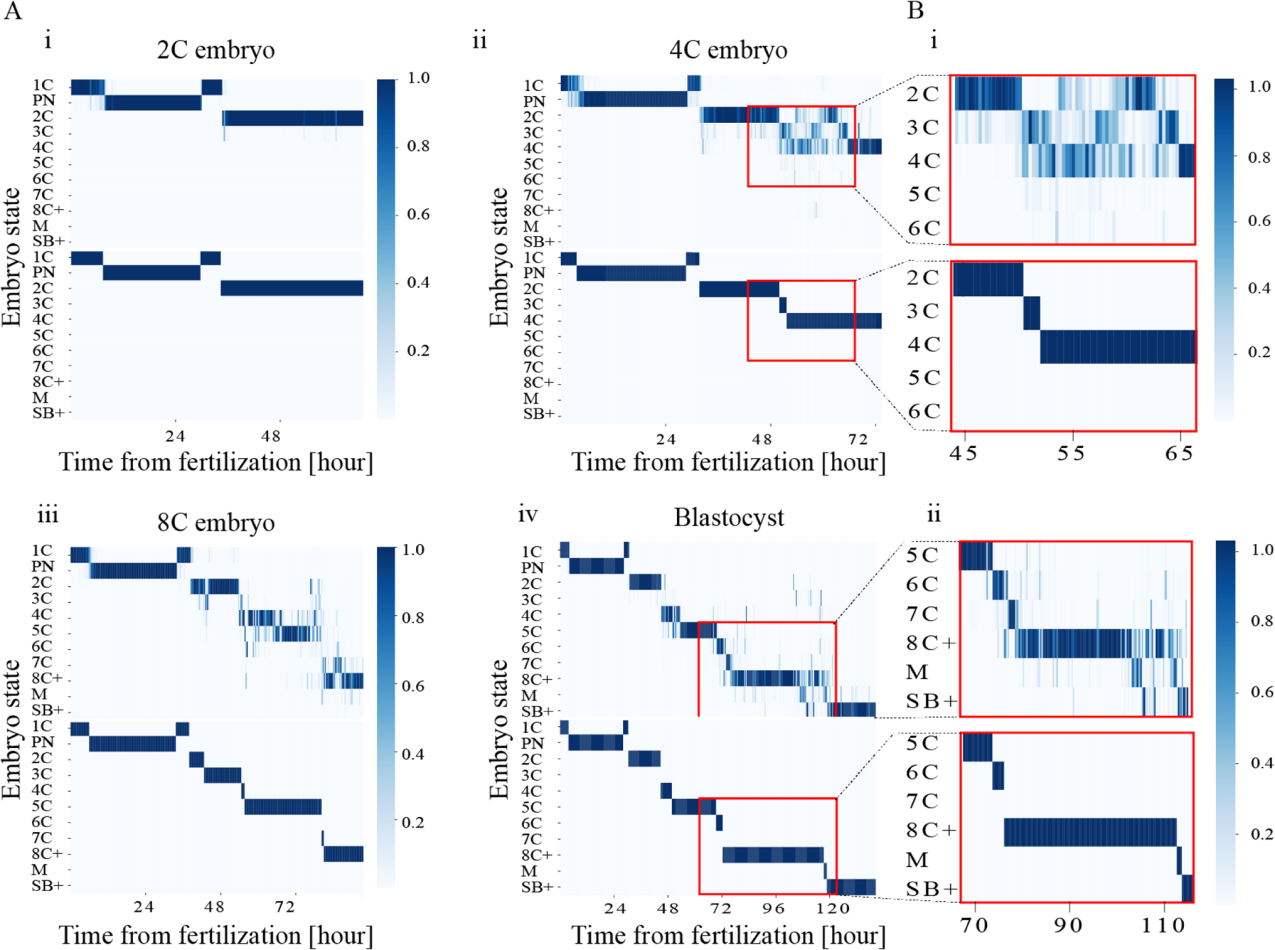
Uncertainty in the assessed embryo state probability vectors is localized to specific developmental regions. **A** Representative ESPM (top) and ESM (bottom) of embryos at (i) 2C, (ii) 4C, (iii) 8C, and (iv) blastocyst developmental states. **B** Zoom-in into the developmental regions of high uncertainty of (i) a 4C embryo, and (ii) a blastocyst. 2C, two-cells; 4C, four-cells; 8C, eight-cells

Using a probabilistic presentation of the embryo states, as presented by the ESPM, we propagate the information that is stored by the uncertainty in each frame (Fig. 4A – top panels). We find that the regions of high uncertainty change between embryos; however, noise tends to be high during the second (3C to 4C) and third (4C to 8C) embryo cleavage blocks and during morula compaction (Fig. 4B). In the process of projection of the ESPM onto single embryo states, as presented by the (ESM), we take into account the temporal neighborhood constrains of the temporal monotonicity of preimplantation development (Fig. 4A – bottom panels and Fig. 4B).

### Classification model performance

**Fig. 5:**
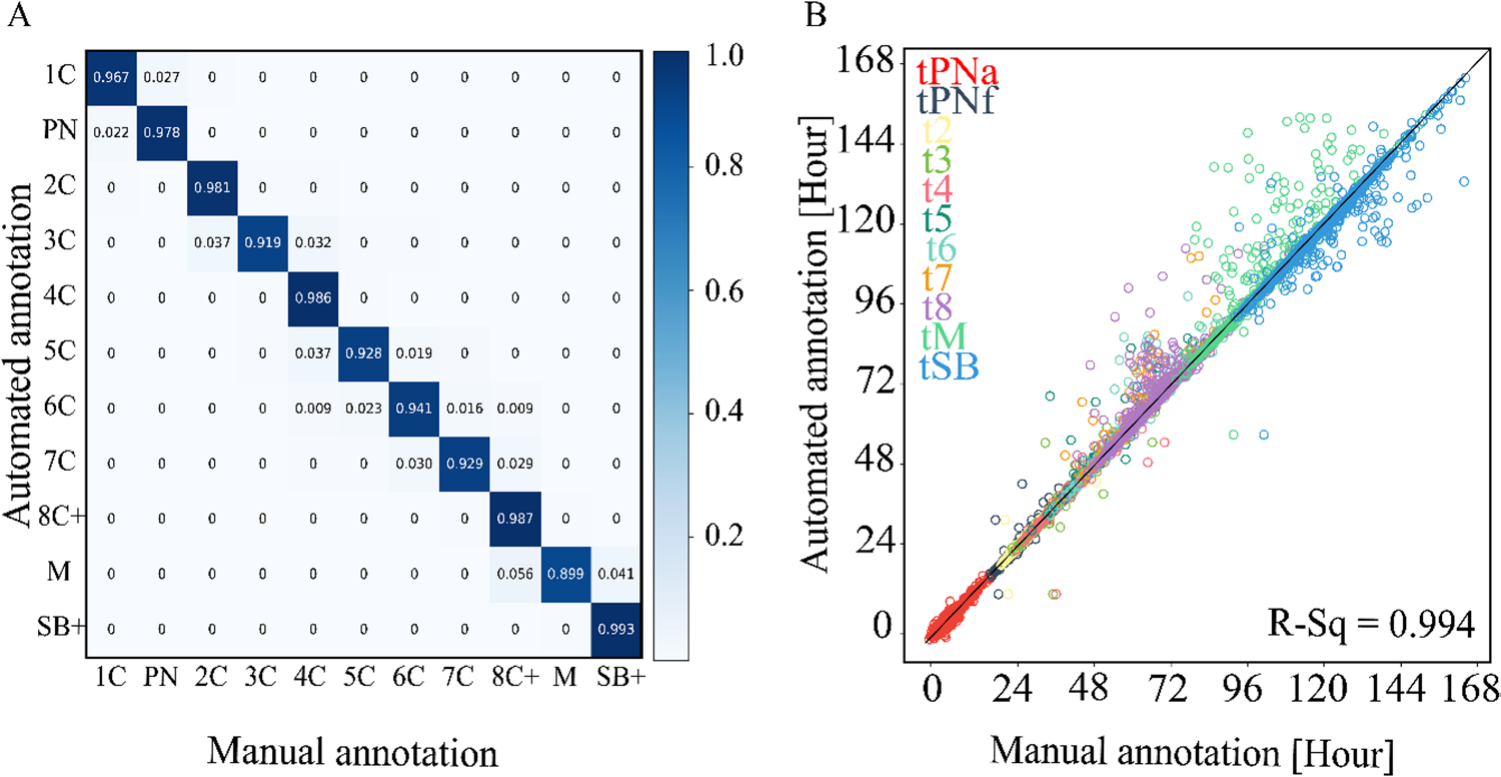
Accuracy evaluation of automated annotation. **A** A confusion matrix presenting the normalized associations between manually-annotated (ground truth) and automatically-annotated (inference) test set embryo frames. For example, 3.7%, 92.8%, and 0.019% of the automatically-annotated 5C frames were manually-annotated as 4C, 5C, and 6C, respectively. **B** The automated prediction of the morphokinetic events correlate with the manually annotated ground truth with R-square 0.994 accuracy. Statics is based on 17,077 morphokinetic events expressed by 1918 test set embryos

To estimate the accuracy in the classification of the embryo states, we calculated the confusion matrix between the ESM and the manually annotated ground truth as averaged across 66,2021 frames of 1918 test set embryos (Fig. 5A). Indeed, most embryo states were inferred in agreement with the ground truth, with 93% precision and 93% recall, whereas disagreements were localized to developmentally-adjacent states. We next address the accuracy of morphokinetic annotation by plotting the predicted versus the manually annotated morphokinetic events of the test set embryos (Fig. 5B). Consistent with the classification accuracy of the embryo developmental states, we obtain 0.994 R-goodness of fit. Scatter plots and linear regressions for each event are provided in figure S2.

The average and standard deviation values of the temporal differences between the automatically and manually annotated events are summarized in Table 4 together with the percentile-wise error distributions. The mean error in the prediction of the pronuclei events (tPNa-tPNf) and the cleavage events (t2-t8^+^) was smaller or comparable with the time-lapse interval (20 min), which sets the minimal sampling error for morphokinetic annotation owing to the discrete nature of video recordings. However, tM and tSB mean errors spanned two time intervals or more, which may be indicative of the inherent ambiguity in the visual determination of start of morula compaction and start-of-blastulation, respectively. These disagreements between manually annotated and automatically annotated events are illustrated by four representative embryos in figure S1. Consistent with the statistical error analysis that we present in Table 4, automated annotation tends to be either in agreement with manual annotation, or to be separated by one or two sequences. Obviously, these four embryos are presented only to provide a visualization of the embryo states that is complementary to the statistical analysis that we performed.

**Table 4:** The average (μ) and standard deviation of the mean (σ) values of the temporal differences between inferred and ground truth annotations were calculated across the specified number of test set embryos. Below this we present the distributions of the temporal differences between automated annotation and manual ground truth per event. The specified percentiles are provided in hours

Events	tPNa	tPNf	t2	t3	t4	t5	t6	t7	≥ t8	tM	≥ tSB
Embryos	1713	1707	1705	1672	1657	1496	1466	1434	1399	1081	1001
μ [hr]	0.07	0.26	0.13	0.08	0.14	− 0.06	− 0.06	− 0.17	− 0.42	− 1.04	0.72
σ [hr]	0.52	0.56	0.38	0.81	0.76	1.01	1.24	1.62	2.59	4.72	3.00
25%	− 0.25	0.25	0.00	0.00	0.00	0.00	0.00	0.00	0.00	− 0.25	0.00
50%	0.00	0.33	0.00	0.00	0.00	0.00	0.00	0.00	0.00	0.00	0.33
75%	0.33	0.33	0.33	0.33	0.33	0.33	0.33	0.33	0.33	0.33	0.66
80%	0.33	0.34	0.33	0.33	0.33	0.33	0.33	0.33	0.33	0.33	0.67
85%	0.50	0.34	0.33	0.33	0.33	0.33	0.33	0.33	0.33	0.33	1.00
90%	0.67	0.50	0.33	0.33	0.33	0.33	0.33	0.33	0.33	0.34	1.67
95%	1.00	0.67	0.34	0.34	0.34	0.33	0.37	0.34	0.67	0.67	4.67
99%	1.67	0.75	0.66	0.67	0.67	0.67	0.67	0.67	1.00	1.34	14.00

**Fig. 6:**
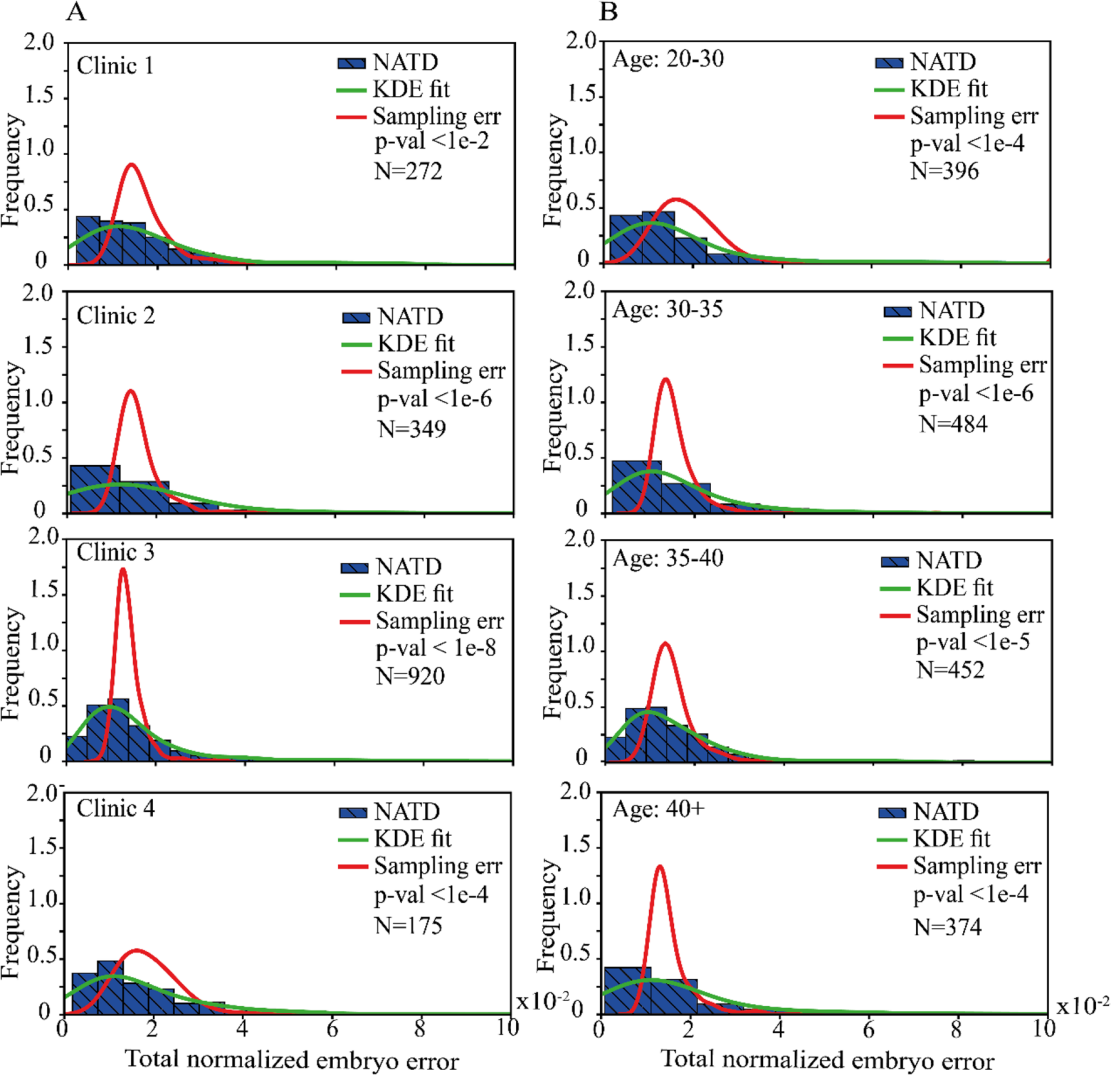
Error analysis of automated morphokinetic annotation of embryos. The NATD histograms provide quantification of the morphokinetic difference per embryo between the automatically-predicted annotations and the manually-annotated ground truth. Histograms and KDE fits are plotted for each data-providing clinic (**A**) and across maternal age groups (**B**). The basal sampling error distributions account for a one time-lapse interval per event between automated and manual annotations. The number of embryos in each cohort is specified. Student’s *t*-test *p*-values are calculated between the KDE fits and the basal sampling error. NATD, normalized absolute temporal difference; KDE, kernel density estimation

Above, we presented the accuracy in the prediction of individual embryo states and morphokinetic events as we evaluated across a large dataset of embryos. However, clinical applications would also require quantitative assessment of the annotation accuracy of the morphokinetic profiles from tPNa to tSB of individual embryos, which will allow consideration of classification generality. To this end, we defined the normalized absolute temporal difference (NATD) between the automated and manual annotations as:

$${E}^{j}=\sum_{i}\left|{A}_{i}^{j}-{M}_{i}^{j}\right|/{M}_{i}^{j}$$

where ${A}_{i}^{j}$ and ${M}_{i}^{j}$ are the automatically and manually annotated morphokinetic profiles, denoted by index event $i$ of embryo $j$. For each embryo, it provides the sum of time differences between the predicted and ground-truth events normalized to time of event. Since differences in the IVF protocols may vary between medical centers and since maternal age is linked with temporal morphokinetic profiles [36, 37], we calculated the NATD histograms as evaluated for test set embryos stratified by clinic (Fig. 6A) and by maternal age (Fig. 6B). The distributions of the basal sampling error per embryo, as determined by one time-lapse interval difference between ${A}_{i}^{j}$ and ${M}_{i}^{j}$ (~ 18 min), are presented. Satisfyingly, we find that the NATD error distributions were broader yet statically-significantly smaller than the basal sampling error. We complement our error analysis by excluding potential confounding contributions due to systematic differences in the automated and manual annotations and in maternal age between the clinics (Fig. S3).

In summary, we report unprecedented accuracy statistics of automated morphokinetic annotation ranging from single-frame embryo state prediction, to inference of population-level morphokinetic events and whole-embryo morphokinetic profiles. Automated morphokinetic annotation is demonstrated to be robust to medical center and maternal age, thus satisfying generality.

### “Big data” analysis of preimplantation embryo development

**Fig. 7:**
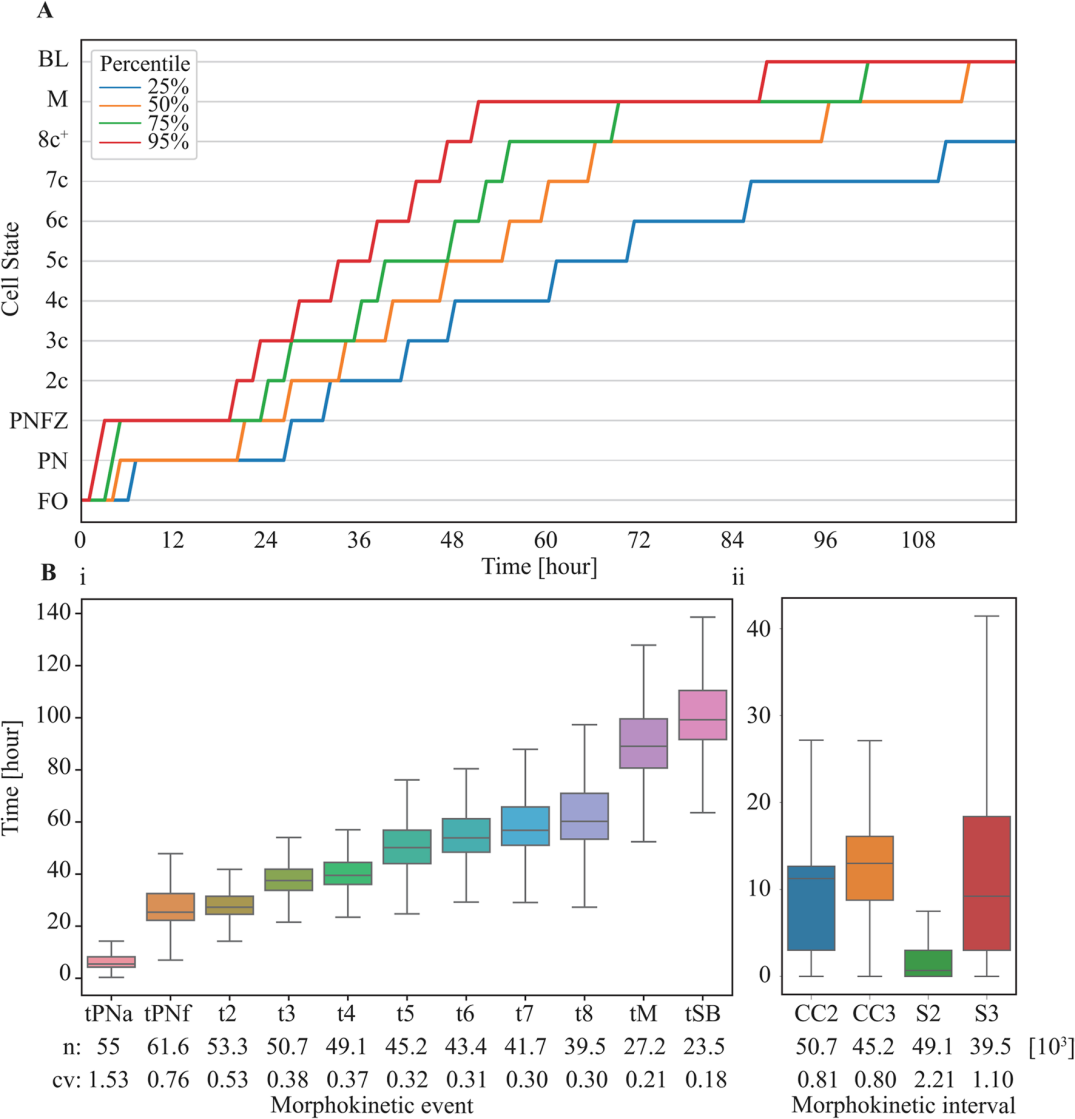
Statistics of preimplantation development of *tens of thousands* of embryos. **A** Embryo state trajectories are evaluated at 1-h resolution from time-of-fertilization to 120 h as depicted for the specified percentiles across 24,644 embryos with 120 h or longer time-lapse recordings. Statistics includes transferred and non-transferred embryos. **B** The temporal distributions were calculated for the specified (i) morphokinetic events and (ii) the CC and CS intervals. The number of embryos and the CV values are specified for each event below the graphs. Analyses are based on automated inference of embryo developmental states and morphokinetic events. Whiskers depict the 5th, 25th, 50th, 75th and 95th percentiles. CC, cell cycle; CS, cell synchronization. CC2 = t3-t2. CC3 = t5-t3. S2 = t4-t3. S3 = t8-t5. CV, coefficient of variation

Automated morphokinetic annotation provides means for analyzing preimplantation development of a large number of embryos to provide statistical characterization, which would have been practically impossible otherwise. To address all preimplantation stages, we annotated 24,644 embryos that have been cultured inside time-lapse incubators for 120 h or more. The generality of our analysis is based on the fact that each of such time-lapse incubator culture plates includes multiple embryos of different developmental potential with no apriority bias of maternal age, clinic, or number of retrieved oocytes per collection cycle. At each hour from time of fertilization to 120 h, each embryo was labeled by its developmental state from 1 (FO) to 12 (BL) and the 25th, 50th, 75th, and 95th percentiles were calculated (Fig. 7A). In this manner, developmentally arrested embryos were accounted for according to their corresponding embryonic state. The 25th percentile is defined by the embryos with the slowest dynamics, which became arrested prior to morula compaction. In comparison, the 50th, 75th, and 95th percentile dynamics reach tM by end of Day-4 (96 h), late Day-3 (68 h), and early Day-3 (51 h), respectively, thus demonstrating the temporal variation between embryos. Complementary to the embryo state dynamics, we calculated the temporal distributions of the morphokinetic events, the morphokinetic cell-cycle intervals, and the morphokinetic cell-synchronization intervals, as defined by the transitions between states (Fig. 7B(i,ii)) [38]. Here, the number of embryos that reached each morphokinetic event decreased with preimplantation development due to prior-arrest of some of the embryos. The temporal dispersion of the morphokinetic events across the embryos, as evaluated by the coefficient of variation, was significant, indicating the inherent heterogeneity between embryos of similar genetic background (denoted in Fig. 7B(i,ii)). Importantly, these temporal variations decreased with preimplantation development, thus demonstrating the developmental convergence of non-arrested embryos.

**Fig. 8:**
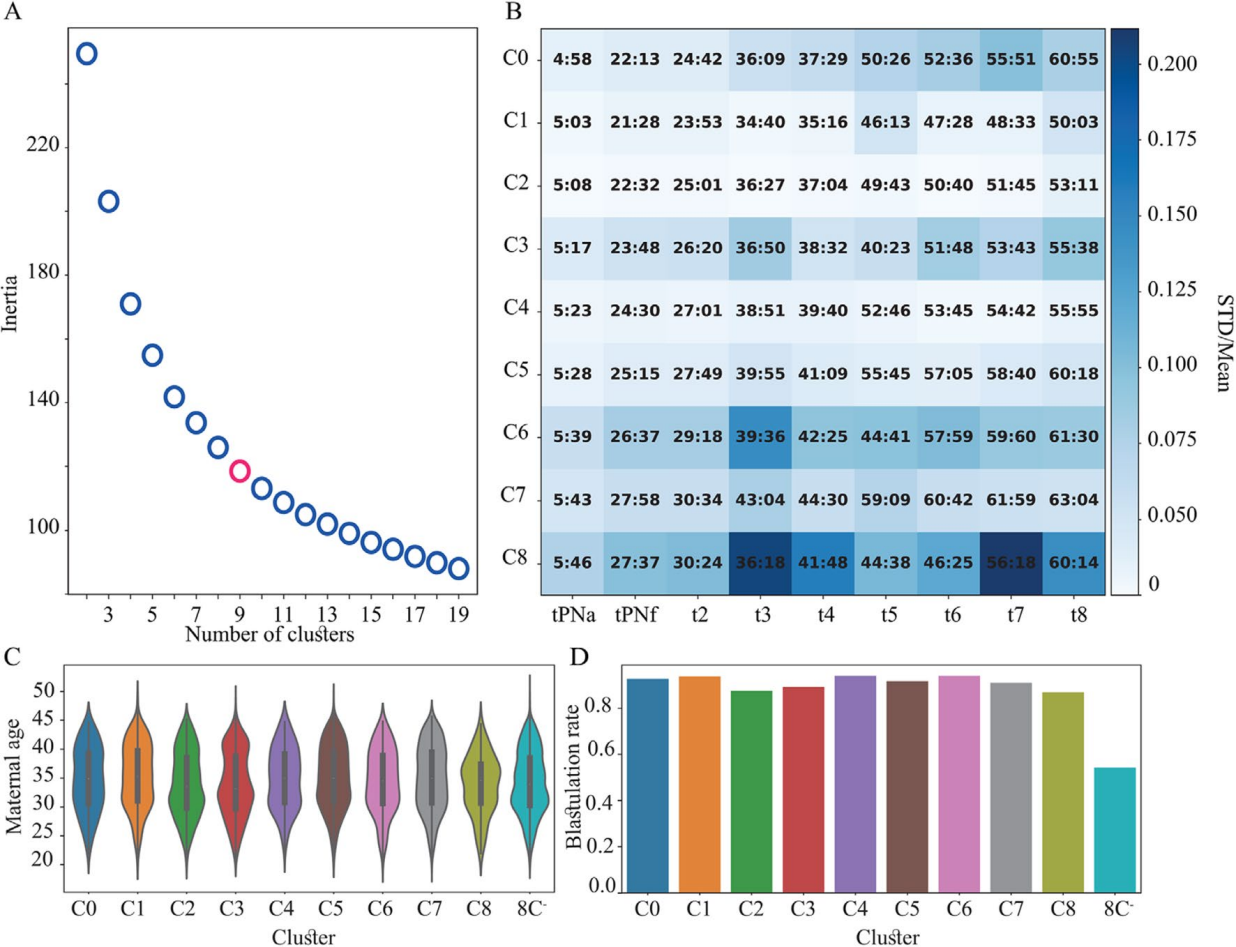
Unsupervised clustering of the embryos. **A** An elbow plot presenting the decrease in variance (inertia) with increasing number of clusters for the unsupervised K-means clustering of high-quality 8C^+^ embryos as performed based on the tPNa-to-t8 morphokinetic profiles. K = 9 clusters was chosen. **B** The clusters C0 to C8 are sorted from fast (C0) to slow (C8) tPNa. **C** The maternal age distributions are indistinguishable across embryo clusters. **D** The C0-to-C8 clusters of 8C^+^ embryos reach start-of-blastulation rate, which is twofold higher than 8C^−^ embryos. Blastulation rates were calculated for embryos in each cluster that were cultured for 120 h or longer. 8C^+/−^: embryos that reached/failed to reach 8-cells stage by 66 h from fertilization

To address embryo-to-embryo variation, we performed unsupervised K-means clustering of the morphokinetic profiles. Since low-quality embryos have poor clinical significance, we included only the high-quality embryos that are generally considered as valid candidates for transfer by excluding the ones that failed to reach 8C by 66 h from fertilization (8C^−^ embryos). We clustered the embryos into K = 9 cohorts based on their tPNa-to-t8 morphokinetic profiles (discarding tM and tSB events), thus allowing high variation while limiting the number of clusters (Fig. 8A). The clusters C0 to C8 are sorted from early to late tPNa (Fig. 8B). The size of clusters C0 to C8 is listed in Table 5 and compared with the cohort of 8C^−^ low-quality embryos that failed to reach 8C by 66 h from fertilization. Importantly, we find that the differences in preimplantation dynamics between the clusters are not associated with differences in maternal age (Fig. 8C) nor in the rate of blastulation (Fig. 8D).

**Table 5:** Number of embryos in each cluster. The excluded embryos that failed to reach the 8C stage within 66 h from fertilization (8C^−^) are pooled together

Cluster	C0	C1	C2	C3	C4	C5	C6	C7	C8	8C^−^
Size	1189	1979	2725	1109	2403	2156	842	1229	527	41,027

**Fig. 9:**
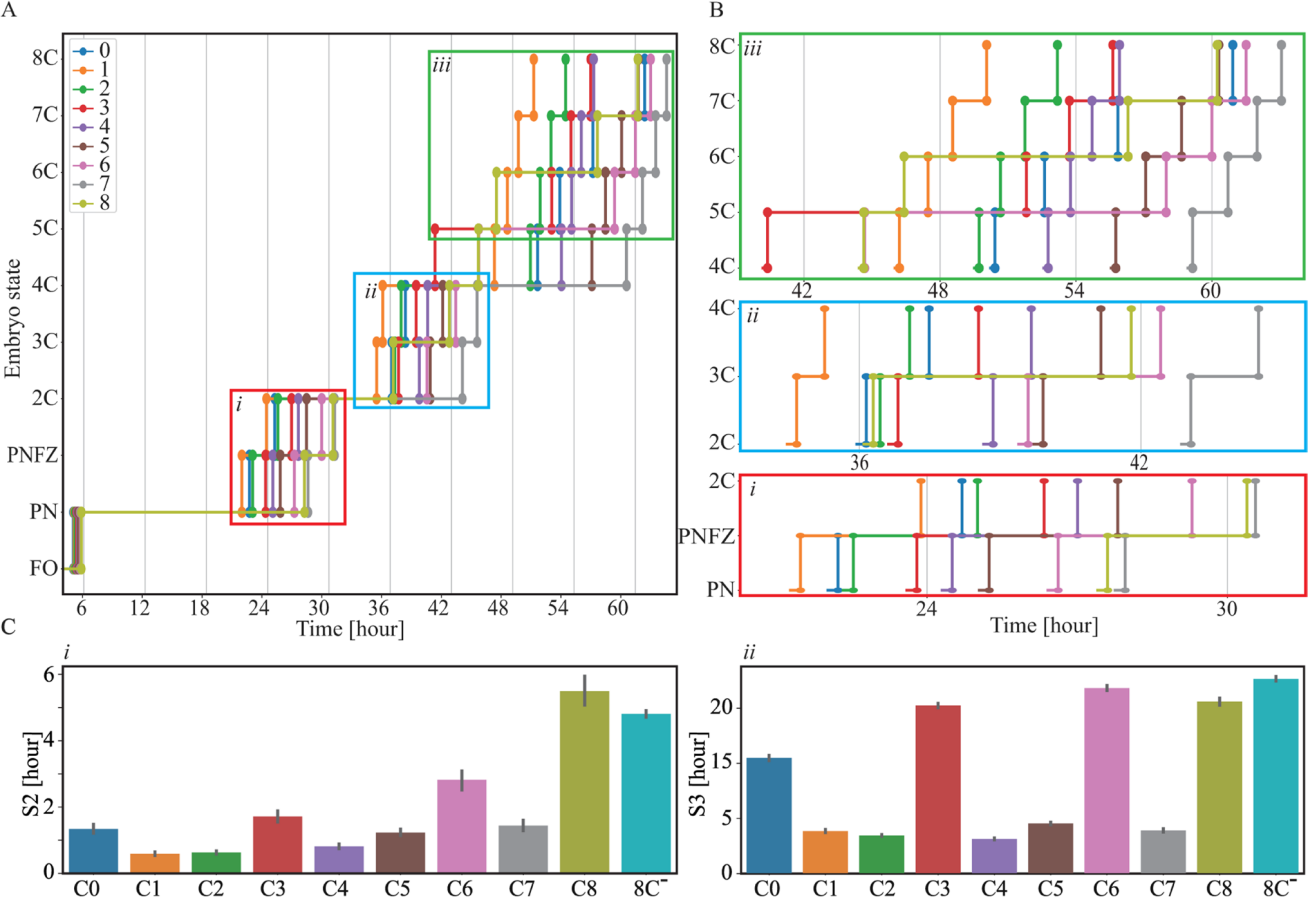
Embryo clusters are characterized by distinctive morphokinetic dynamics. **A** Contours of the average morphokinetic events are shown for clusters C0 to C8 and the cohort of low quality 8C^−^ embryos. **B** Zoom-in diagrams of the first (i: PN to 2C), second (ii: 2C to 4C) and third (iii: 4C to 8C) blastomere cleavage blocks differentiate between developmentally slow and fast preimplantation dynamics. **C** The average cell synchronization intervals of (i) the second blastomere cleavage round (S2 = t4-t3) and (ii) the third blastomere cleavage round (S3 = t8-t5) are shown for C0 to C8 clusters and compared with 8C^−^ embryos. Error bars depict standard deviation

Clustering analysis reveals distinctive morphokinetic patterns that are characteristic of embryo subtypes (Fig. 9A). To characterize preimplantation dynamics, we consider the first, second, and third embryo cleavage blocks (Fig. 9B). All clusters maintain their order from fast to slow during the first blastomere cleavage round (PN to 2C). However, C0 embryos entered the first blastomere cleavage round early but completed the third blastomere cleavage round late. Next, we calculated the second (S2 = t4-t3) and third (S3 = t8-t5) cell synchronization intervals, which quantify the degree of mitotic synchronization between the blastomeres in the corresponding cleavage blocks (Fig. 9C). C3, C6, and C8 embryos are the least synchronized. With respect to the third mitotic cycle, the synchrony of these clusters is as poor as the low quality 8C^−^ embryos.

### The developmental potential of the embryo clusters

**Fig. 10:**
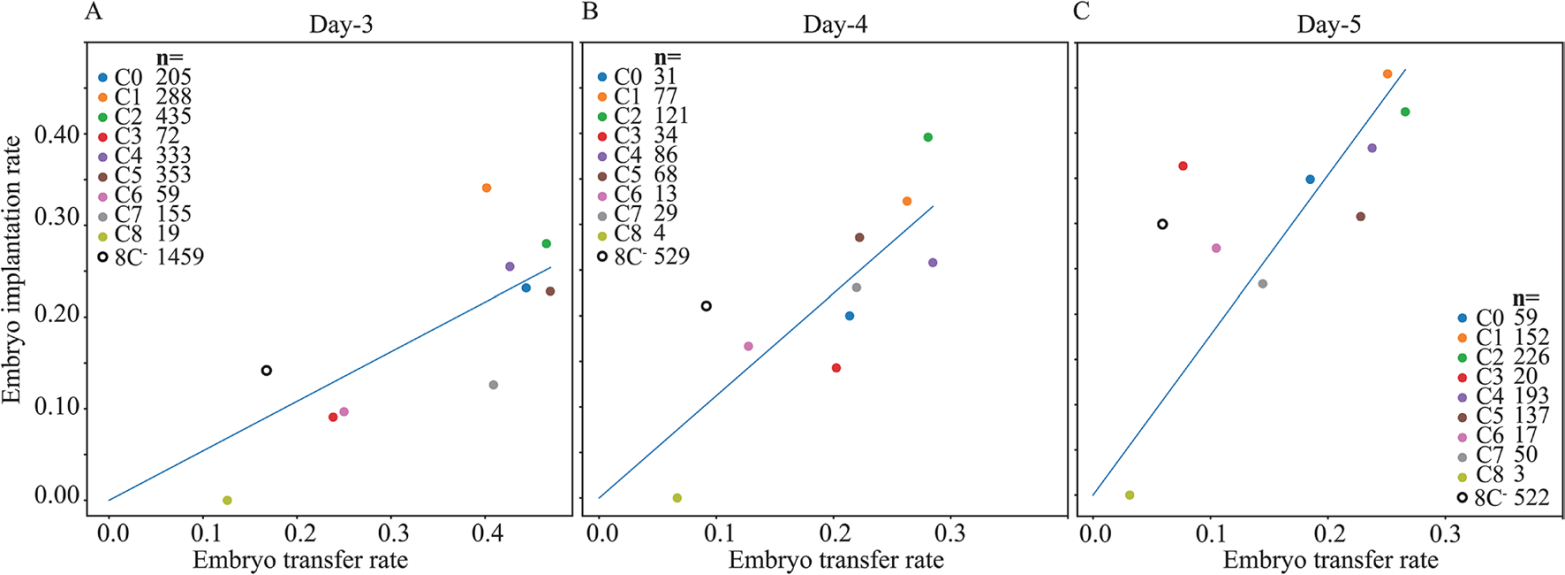
Transfer and implantation characteristic of embryo clusters. Average implantation rates are plotted as a function of the average transfer rates of clusters C0 to C8 embryos and 8C^−^ embryos (at 66 h from fertilization). Implantation-versus-transfer rates are plotted for **A** Day-3 cleavage stage embryo transfers (66 to 74 h), **B** Day-4 embryo transfers (74 to 110 h), and **C** Day-5 blastocyst transfers (110 to 125 h). The number of transferred embryos is specified in the legends

The rate of embryo transfer into the uterus reflects the developmental quality of the embryos as estimated by the clinicians. To assess the relationship to the developmental potential of the embryos, we plot the average implantation-versus-transfer rates of the clusters of Day-3 (Fig. 10A), Day-4 (Fig. 10B), and Day-5 (Fig. 10C) transferred embryos, and include 8C^−^ low-quality embryos. As expected, the implantation rates of Day-5 transferred embryos are highest and of Day-3 transferred embryos are lowest. The decrease in embryo transfer rates between Day-3 and Day-5 transfers is due to the decrease in the number of transferred blastocysts per cycle. Despite the fact that the cluster distributions of the available embryos per oocyte collection cycle are not specified, the average implantation rates are positively correlated with the transfer rates, thus conforming the capacity of morphokinetic profiling in predicting embryo quality. C1 and C2 clusters consistently show the highest implantation rate and high transfer rates. C3, C6, and C8 clusters have the lowest implantation rates and low transfer rates during embryo cleavage (Day-3) and morula compaction (Day-4) stages, which is consistent with the poor cell cleavage synchronization of these embryos during the third mitotic cycle as shown above (Fig. 9C). Hence, our unsupervised clustering analysis confirms the role of S3 as a marker of embryo developmental potential [17, 39]. We note that blastocyst selection for transfer on Day-5 significantly improves the implantation rates of C3 and C6 clusters (C8 embryos have zero implantation rate).

## Discussion

Temporal profiling of the morphokinetic events has been demonstrated to support the evaluation of the developmental potential of embryos and improve implantation rates by allowing the selection of the highest-quality embryos for transfer [40]. Computationally, morphokinetic annotation provides means to effectively reduce the dimensionality of the video representations of the embryos from ~ 100 Mb to ~ 100 bytes, thus preventing overfitting and improving accuracy given a finite dataset and computational power. Despite its now-established efficacy, the utilization of morphokinetically-based embryo evaluation algorithms in clinical settings is hindered by the substantial workload that is required for performing manual annotation, and by potential intra- and inter-observer variations [26–29]. Computer-based automated algorithms are required in order to lift these hurdles and facilitate the utilization of morphokinetic-based decision support tools in clinical settings.

To date, various machine learning methodologies were designed that can automatically annotate blastomere cleavage [30–32] as well as morula compaction and blastocyst expansion [34]. Recently, automated annotation spanning the entire course of preimplantation development was reported, which can potentially support embryo selection for Day-5 transfers. Feyeux et al. incorporated image processing tools to reach 92% accuracy whereas Leahy et al. reports 87.9% accuracy by training multiple CNNs [33]. For both cases, clinical utilization as a stand-alone automated decision-support tool would likely require improved accuracy [41]. To improve accuracy, we assembled an expansive retrospective dataset to train a CNN for assessing the embryo state in each frame while allowing a probability-weighted superposition of multiple states. The individual states are determined via monotonic regression, thereby integrating morphokinetic dynamics across a finite temporal vicinity around each event under temporal monotonic constrain. In this manner, we demonstrate fully automated annotation from tPNa to tSB with unprecedented accuracy (R-square 0.994), whose error distribution is largely generated by the discrete nature of time-lapse imaging.

Multiple combinations of morphokinetic events and intervals have been selected by various classifiers for evaluating the developmental potential of embryo [42]. In the case of Day-5 transferred embryos, tSB was retrospectively shown to discriminate between high quality (tSB < 96 h) and lower quality (tSB > 96 h) embryos that were selected for transfer based on morphological criteria and assessed via implantation outcome [43]. However, other algorithms also include events that follow tSB [44]. Late events include the time of full blastocyst formation (tB) and the time of expanded blastocyst (tEB). tB refers to state in which the blastocoel is filling the embryo with < 10% increase in diameter [45], or alternatively the frame in which a crescent-shaped area began to emerge from the morula [38]. tEB refers to the state in which the blastocyst’s diameter increases by > 30% concomitant to initiation of zona thinning [46]. Evidently, these definitions of tB and tEB are morphologically complex, which should be considered for practical purposes. The latest event that our algorithm automatically annotates is tSB. However, expanding automated annotation to include additional events is likely feasible provided that suitable manually annotated datasets are available for training.

In this study, we investigated embryo-to-embryo variations among morphokinetic profiles using automated annotation. A total of 24,644 embryos were cultured and recorded for over 120 h. To ensure statistical robustness, we excluded low-quality embryos and only analyzed high-quality embryos that were 8C + at 66 h from fertilization and are considered as valid candidates for transfer. Using unsupervised K-means clustering of 14,159 8C + embryos, we identified nine subtypes with distinctive morphokinetic dynamics, including fast and slow developing embryos, as well as embryos that start fast and finish slow. Notably, a recent report performed self-supervised clustering of the time-lapse video files in order to assess embryo viability, which demonstrates the utility of entire frame-wise clustering approaches [47]. The maternal age distributions of the clusters overlapped and were statistically indistinguishable from low-quality 8C- embryos, supporting the notion that maternal age may affect the size of the oocyte collection cycle but not the inherent heterogeneity of embryos within each cycle [48]. We found that similar blastulation rates were exhibited by the clusters, which is consistent with the high developmental quality of the embryos. However, the implantation rates of the clusters varied, which is indicative of the degree of association between the morphokinetic properties of the embryos and their developmental potential. In particular, three clusters that were characterized by low implantation rates were distinctively characterized by poor synchronization of the third mitotic cell cleavage cycle, thus defining a potential potent marker of embryo quality.

Following this retrospective study, clinical implementation of automated annotation will require its employment and validation in prospective studies. Below, we present in conceptual terms an optional design for a clinical study that is expected to demonstrate safety and clinical utility while satisfying regulatory requirements and overcoming ethical limitations. A multicenter prospective embryo transfer study will include a nonselection arm followed by a controlled and randomized trial in healthy patients [49]. No age or ethnic restrictions should be applied for preliminary screening. The selection of embryos for transfer will be performed according to an established policy that employs a regulatory-approved morphokinetic-based decision support tool. The IVF protocols and the decision making process for determining the day-of-transfer and number of transferred embryos per cycle will not be modified. For the nonselection study, automated annotation will be performed blindly parallel to manual annotation. The implantation potential of the embryos will be based only on the latter and the embryos with the highest predicted developmental potential will be selected for transfer. Once the implantation outcomes of the transferred embryos are determined, the automated annotations will be unblinded, their implantation potential will be evaluated using the same morphokinetic criteria, and the corresponding predictive value will be calculated and compared with the existing policy. Unless the predictive value that is generated by automated annotation is significantly inferior to the current policy, a randomized controlled trial will be performed next. The embryos in the treatment and the control groups will be annotated via automated and manual annotation, respectively, their implantation potential will be predicted, and the embryos for transfer will be selected as described above. The clinical benefit of automated annotation will be calculated by comparing the implantation rates of the control and treatment groups. To mediate ethical constrains, a preliminary study will be excluded to a subgroup of high-quality embryos that will be prescreened based on their predicted developmental potential. Given the obvious advantages of automated morphokinetic annotation over manual annotation, prospective studies are required to demonstrate predictive value and clinical benefit that are either comparable or exceeding manual annotation. In addition to overcoming the obvious limitations of manual annotation in the busy laboratory settings and eliminating inter-observer and intra-observed variations, clinical utilization of automated morphokinetic annotation will provide standardization of embryo selection protocols, improve implantation and live-birth rates while shortening time-to-pregnancy and support single embryo transfer policies.

## Supplementary Information

Below is the link to the electronic supplementary material.

Supplementary file1 (PDF 2798 KB)

## Data Availability

The copyrights of the code are owned by Yissum–the technology transfer company of The Hebrew University of Jerusalem. Requests can be sent to A.B. The clinical data are owned by Hadassah Medical Center and by Clalit Health Services. Restrictions apply to the availability of these data, which were used anonymously under ethical agreements with each clinic separately for this study, and so are not made publically available. Access requests can be directed to A.B.M. (Hadassah Medical Center), Y.O. (Kaplan Medical Center), I.H.V (Soroka University Medical Center), Y.S. (Women's Hospital, Rabin Medical Center).
