# Supplementary material for: Delineating the heterogeneity of embryo preimplantation development using automated and accurate morphokinetic annotation
Source: J Assist Reprod Genet. 2023 Jun 10;40(6):1391–406. doi: 10.1007/s10815-023-02806-y (PMC10310622; doi:10.1007/s10815-023-02806-y)
Supplement: Supplementary file 1 — Supplementary file1 (PDF 2798 KB) [file 10815_2023_2806_MOESM1_ESM.pdf]

1 SUPPLEMENTARY MATERIAL

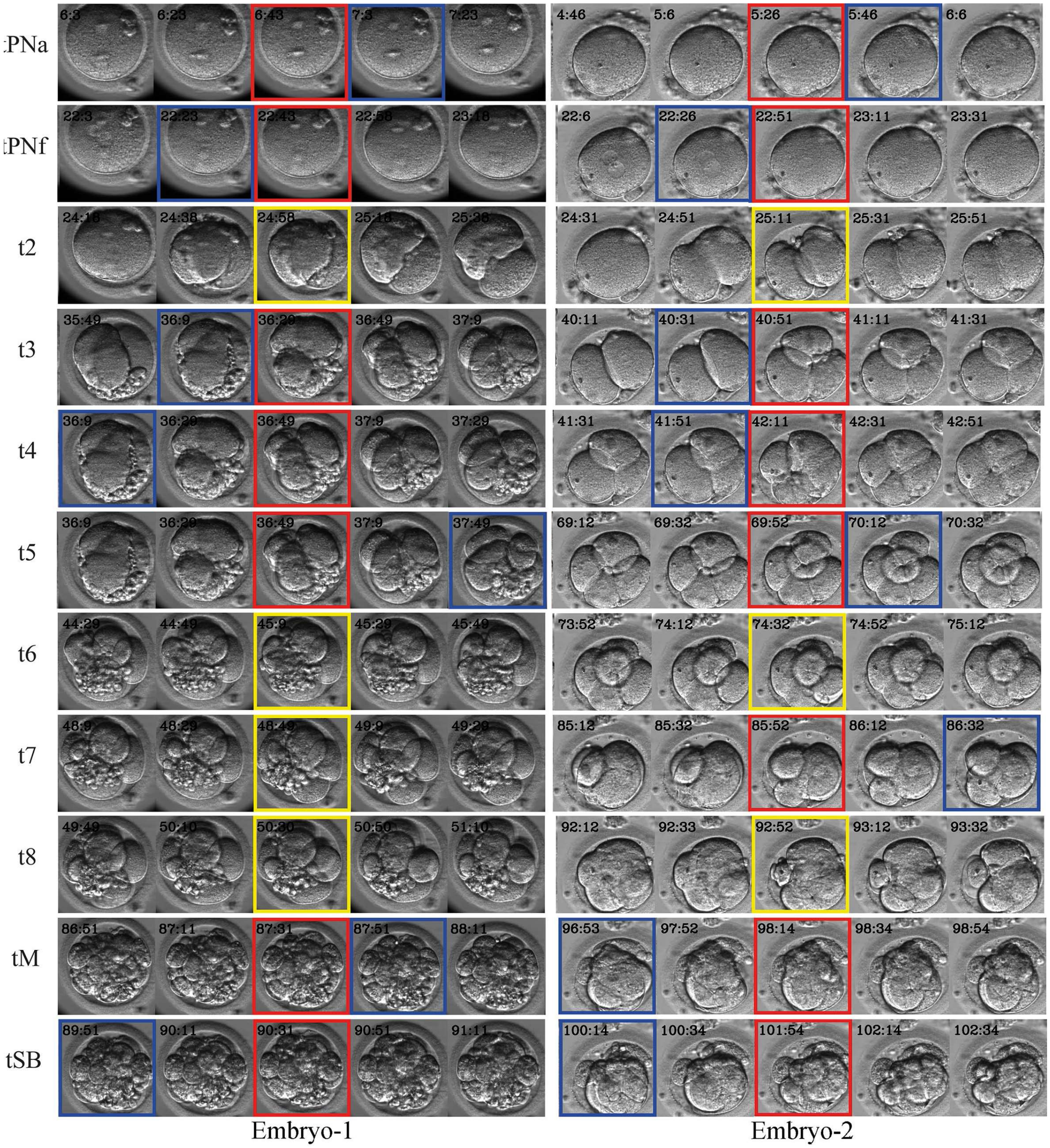

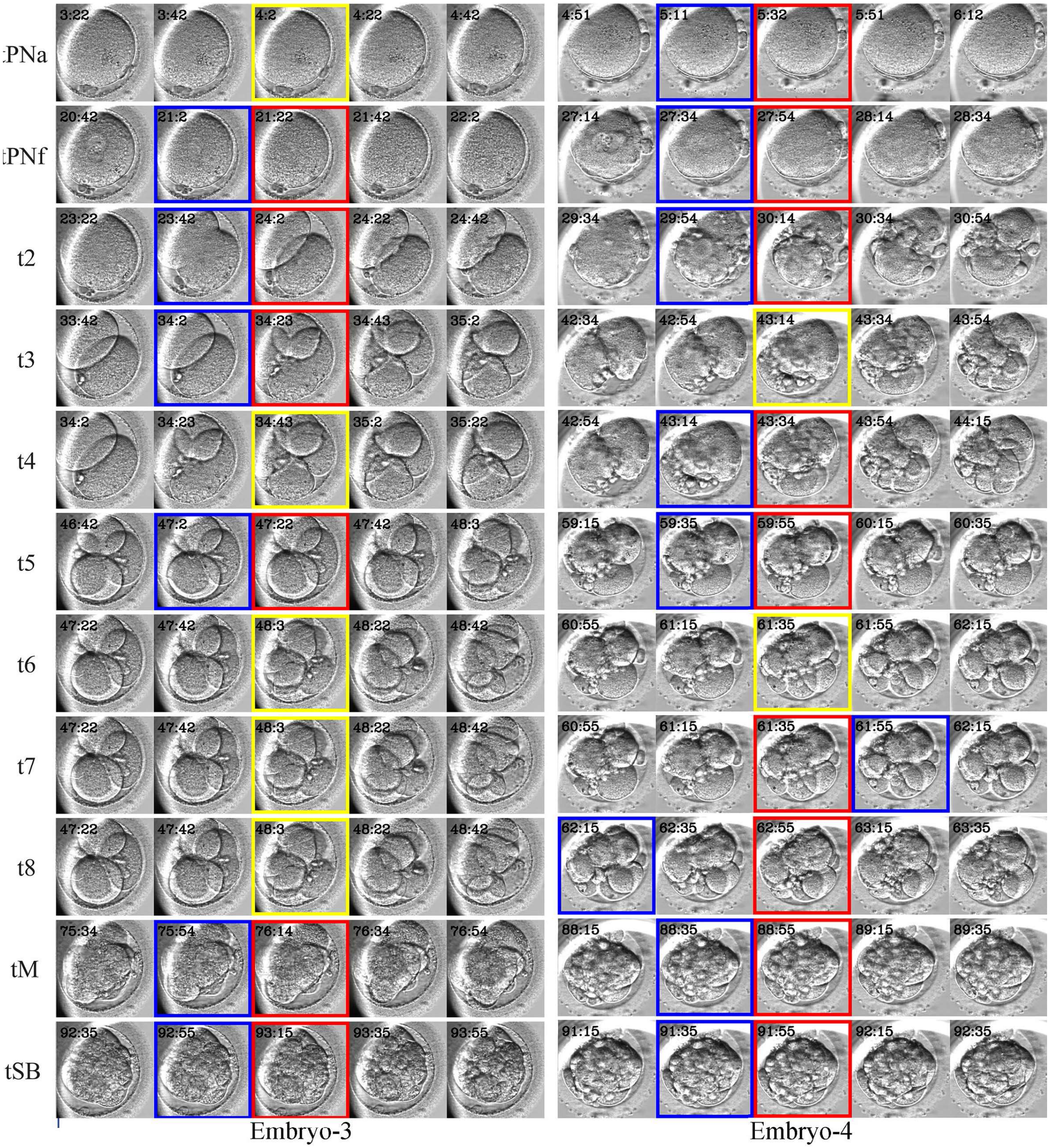

Figure S1 | Automated morphokinetic annotation. A comparison between the automated morphokinetic annotations and the manual ground truth is demonstrated for four representative embryos. Five consecutive frame strips (rows) are centered around each ground truth event (central column). Manually-annotated frames are marked by red frames. Automatically-annotated frames are marked by blue frames. Manually and automatically co-annotated frames are marked by yellow frames. Time stamps are specified ([HH:MM]; top-left).

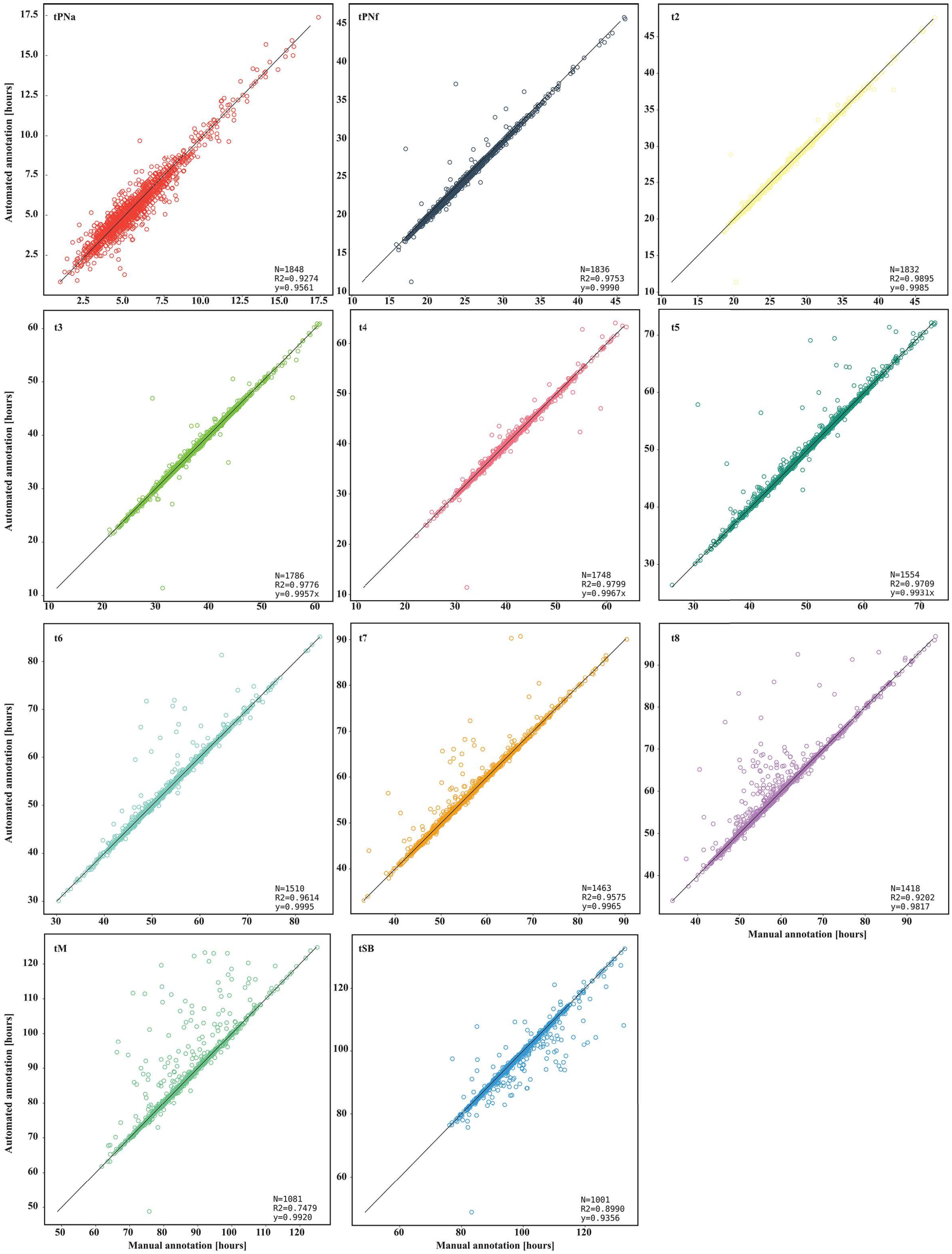

Figure S2. Correlations between manual and automated annotations. Automated annotations are plotted versus manual annotations of tPNa to tSB event and degree of correlation is assessed via linear regressions. The number of embryos (N), R-square goodness of fits, and the fitted parameters are specified.

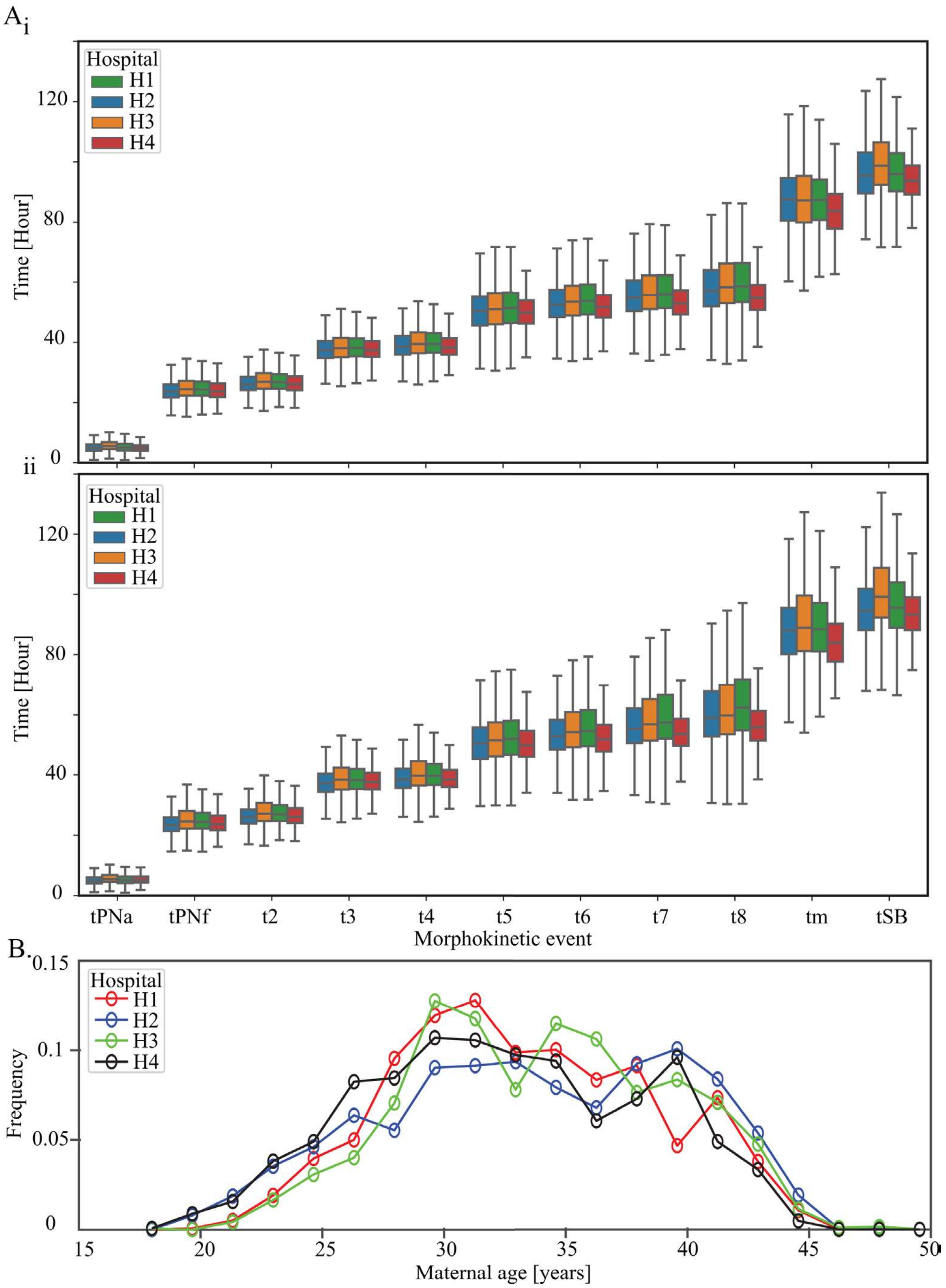

Figure S3 | Morphokinetic annotations and maternal age across data-providing hospitals. (A) Temporal distributions of (i) manually annotated and (ii) automatically annotated morphokinetic events across clinics. Whiskers depict 5%, 25%, 50%, 75% and 95% levels. (B) Normalized histograms of the maternal age distributions across clinics.
